# A SWOT analysis of the EBOD exam 2019

**Published:** 2019

**Authors:** Stanca Horia Tudor

**Affiliations:** * Chief of the Eye Clinic, “Prof. Dr. Agrippa Ionescu” Clinical Emergency Hospital, Bucharest Ophthalmology Department, “Carol Davila” University of Medicine and Pharmacy Bucharest

I have been practicing ophthalmology for 20 years, so I could say that I am already playing for the “old boys” team in this field. I have always had a strong interest in improving education for ophthalmologists and I have meticulously studied some European systems and also the American one. I have collected several recognitions of my professional education and clinical abilities during these years from some prestigious institutions like EVRS, ICO, ISRS, and AAO, but I have missed the FEBO title. Was this one so important to me, taking into account my daily activities as an ophthalmologist in Romania? Of course not, but I have noticed a positive trend in getting the “European” recognition in the last years so I decided to get it. There were two ways for me to obtain this title: by participating as an examiner to European Board of Ophthalmology Diploma (EBOD) Exam yearly for at least three sessions or by having the examination as a regular “young” candidate. I chose the last option, so I went to Paris to take the EBOD exam in May 2019. Why I decided to be examined? Because I thought that it would be more dynamic and interesting to be a player, instead of a referee or a coach, and because I wanted to feel the same as the young doctors who take this exam, just in order to better understand their demands. Two of my trainees who came to get an objective validation of their training have accompanied me.

My first impression about the registration procedure was good. Everything was well set and I received all the information I needed to be present in time for the exam. I had been informed about the schedule of my examinations and the locations of the rooms I had to visit for the written part and for the oral one. When I arrived to the Congress Palace the high number of participants impressed me, but I should mention that EBOD is equivalent to the national exam in Switzerland, Belgium, France, Austria, and Ireland, so the bulk of the people were from those countries. Germany had also sent many candidates. The examination rooms’ location was easy to find and all the participants had a unique number, which accompanied them all the time, including on the successful or failed candidates lists (these lists consisted of only the identification number and no names).

The structure of EBOD exam is detailed on the EBO website, so I will not insist on it, but I have to underline that for the written part, there are 52 MCQ questions that “cover any basic science, medical or surgical topic relevant to the practice of ophthalmology” and the candidate should answer to them in two hours. The MCQs consist of a stem followed by five statements, each of which needs to be judged as “true or “false” and you may opt for “do not know” if necessary, but I do not recommend the use of this option too frequently because it is a statistical trap. Consequently, 260 answers will be required. By the way, the examination language is English. The written examination is followed by the oral one (Viva-Voce exam). Each candidate should proceed to a certain designated room at a specific time. There are four tables positioned in a square and the candidate should step in its middle. There are two examiners at each table, facing each candidate for 15 minutes, and the candidates rotate to cover all four tables in 60 minutes. The oral examination is an interview with questions supported by imaging resources (notebooks facing the candidate). The table groups (8 examiners) mentioned before cover four sections grouped as it follows: 

A. Optics, Refraction, Strabismus, Pediatric ophthalmology and Neuro-ophthalmology;

B. Cornea, External Diseases, Orbit and Ocular Adnexa;

C. Glaucoma, Cataract and Refractive Surgery;

D. Posterior Segment, Ocular Inflammation, and Uveitis.

The dynamics of this kind of exam is intense and the candidate should be much focused in order to be able to switch his attention to many different topics. Most of the examiners seemed to be supportive to candidates, according to their instructions. Some of them were fluent in English, some of them were not, but it did not seem to me that this fact affected the candidates’ evaluation. The EBOD exam started at 8.30 in the morning with the written part and the last candidates finished around 18.45 in the evening with the oral examination. There is a huge turnover of people, and the organization should be perfect in order to respect the time coordinates and the evaluation required criteria. In addition, it was!

Why I am entitling my paper “A **SWOT** analysis of EBOD exam 2019”? Because, according to Wikipedia, the SWOT analysis (or SWOT matrix) is a strategic planning technique used to help a person or organization identify the strengths, weaknesses, opportunities, and threats related to business competition or project planning. Users of a SWOT analysis often ask and answer questions to generate meaningful information for each category to make the tool useful and identify their competitive advantage. The name is an acronym for the four parameters the technique examines:

**- Strengths:** characteristics of the business or project that give it an advantage over others.

**- Weaknesses:** characteristics of the business that place the business or project in disadvantage relative to others.

**- Opportunities:** elements in the environment that the business or project could exploit to its advantage.

**- Threats:** elements in the environment that could cause trouble for the business or project.

 Strengths and weakness are frequently internally related, while opportunities and threats commonly focus on the external environment.

 The SWOT analysis has been used in community work as a tool to identify positive and negative factors within organizations, communities, and the broader society that promote or inhibit successful implementation of social services and social change efforts. That is why I chose this kind of analysis for the EBOD examination, which mainly addresses the young ophthalmologists’ community from all over Europe, in order to achieve a common platform of knowledge and training for everyone involved and to continuously improve these items by having designated professional requests.

 So let us discuss each of these items according to my own experiences:

**- Strenghts:** I think that the main strength of the EBOD exam is the excellent organization. It is a very difficult task to properly manage an exam with several parts and many candidates and you need an excellent staff, a good location, an “on the clock” program, and obviously enough money to support all of these. EBO seemed to have succeeded to accomplish each of these factors. The EBO staff and the volunteers worked perfectly together. The Paris Congress Palace offered a quite adequate space for having the examination. The program for both exams was perfectly set. I cannot discuss about funding because I do not have data, but I can mention that the fee to sit the EBOD examination was moderate and the organizers tried to properly accommodate the candidates with water and good conditions to have the exam. I cannot end the strengths’ discussion without mentioning the examination itself. Both parts, the written and the oral one, had a very good design. The MCQs were nicely balanced through the almost entire field of ophthalmology and each statement was judiciously elaborated according to the psychometrical tests requirements. Probably the Viva Voce exam had been created having in mind two items: objectivity and wide covering of themes. Having 8 examiners for each candidate is a strong and valuable tool to objectively evaluate the applicants and the four sections, grouped at four tables I mentioned before, they covered the comprehensive ophthalmologists’ needs. The clinical cases were suggestively illustrated and the questions seemed logical and reasonable to me. There is a section on the EBO’s website, “Statistical Monitoring & Validation of EBOD”, which clearly explains the rationale behind all the examination’s steps, in order to be reliable and stable over the years.

**- Weaknesses:** Whenever an objective analysis is made, some weak parts should also be reflected. Talking about the EBOD examination, I think that there are some not very strong parts regarding the human factor and the time. The human factor is the most difficult to manage and, for such a huge exam a high number of examiners is needed, who probably come as volunteers, and this involvement has to be appreciated. Obviously, not all the examiners have the same training and the same kind of judgement and that is why the EBO Committee should try to create more homogeneity among the examiners, by asking more precise tasks and by controlling the oral examination progress more rigorously. Probably, the examiners should be rotated 6 rounds of one hour each and they need some breaks, otherwise the fatigue will affect their objectivity and their full involvement in the evaluation of each candidate. The time factor interacts with the human one in this part, because everything should be done within 10-11 hours and the EBOD exam should be closed at the end of the day. The proper selection of examiners is tough and probably difficult to do, because many people are needed. My suggestion is that the ophthalmologists with quite enough clinical experience, and who have successfully overcome the EBOD exam could be the best candidates as examiners. Maybe other doctors selected as examiners should have a more detailed training about the objectives, the needs, the principles, and the right tools of a proper examination in accordance with the EBO requirements. Moreover, supervising the examiners by others, more experienced, during the exam, could increase the quality of the evaluation. I noticed that there were supervisors in the examination rooms, but I think that they should directly and randomly observe some of the tables/ examiners in order to validate the assessment quality. All the clinical cases could have an agreement from a higher-level committee and the main questions to candidates for each case could be confirmed.

**- Opportunities:** Probably the main opportunity to the EBOD exam is to set the standards of training in ophthalmology all over the Europe and to become the equivalent of the national exam for more European countries. The excellent organization and the quite high objectivity of this examination are extremely important to convince the ophthalmologists involved regarding the necessity of perpetuating and developing it more extensively in order to become a real validating certification of theoretical and practical skills in ophthalmology. By collaborating with the International Council of Ophthalmology (ICO), a new kind of worldwide recognition as an ophthalmologist could be created. Nowadays, young doctors from Europe attend either the EBOD exam or the ICO (in two steps) or even both, but only EBO rewards the successful candidates with a very distinctive title – Fellow of the European Board of Ophthalmology (FEBO) and offers a quite impressive diploma. I am sure that in the next years, the EBOD exam will grow even more, because there highly trained people need to have a more extensive (international) validation. The FEBO title could guarantee the quality of a certain ophthalmologist training in the next years and could offer him the possibility to be hired anywhere all over the Europe without any constraints. 

**- Threats:** Probably, the main threat for the EBOD exam is to grow too much and too fast in the next years and the quality of evaluation could be affected because of insufficient logistics (enough people, proper location, adequate funding sources). The European legislation could help and protect this kind of European validation for specialists and some forms of funding from the European agencies would be of great support in improvement and development. Despite the European trend to share the same values and principles in every corner of the “old” mainland, there is a strong desire in many countries to preserve their cultural specificities and this is completely understandable. Education is a cultural issue and the Universities and the teaching institutions from different European countries could be quite reluctant in abandoning their evaluation programs in favor of the EBO’s one. I think that besides this kind of validation (EBOD exam), which is not mandatory, the EBO Committee should militate and push more vigorously to have similar principles of education for ophthalmologists all over the Europe. Setting the standards for the residency programs (lengths, curriculum, and logbook) could be a major step in improving the ophthalmologists’ training and creating an educational homogeneity. The ICO has a fantastic program regarding the residents’ education and, collaboration between EBO and ICO in this matter would be more than productive. The accreditation and certification concepts are extensively explained in principles of education textbooks. Theoretically, the accreditation of the teaching centers should precede the certification of the people trained out there, but in real life, it is much more complicated, so these two items should probably go together in order to get some positive results and not to have complete bottlenecks. Having residency-training centers, which share the same methods and curriculum all over the Europe would be more appropriate in having specialists who are successfully certificated by a unique supranational exam.

 This kind of SWOT analysis could be useful to provide directions to the next stages of the change or improvement process of the EBOD examination. The EBOD examination is a great test and I tried to identify some of its strengths and opportunities. Any progressing process is continuously changing, so, for sure, different transformations and an evaluation of weaknesses and threats from a neutral part could be beneficial in the next years.

 I decided to write this paper because I have a special interest in education, being a teacher, and I was positively impressed by the organization of this impressive event, the objectivity and clarity of the examination methods, the high number of participants from all over the Europe. For sure, the EBOD exam is the biggest test for ophthalmologists I have ever seen, succeeding to gather more than 700 candidates and 500 examiners from the entire continent (these numbers were approximated) in the same place and the same time. Since I came back from Paris, I have strongly started to influence my trainees to participate to the EBOD Examination in the next years and I have partially changed my training methods in order to better prepare the residents for the EBO requirements. Despite the fact I am not young anymore and I am not so eager to have countless examinations, this exam in May 2019 in Paris gave me a tasty flavor, because by passing it one can have an objective validation of excellence in ophthalmology. The EBOD examination was designed to assess the knowledge and clinical skills requisite to the delivery of a high standard of ophthalmology care both in hospitals and in independent clinical practices and I encourage every ophthalmologist in Romania interested in having another kind of professional assessment and validation to go for it confidently.

**Senior Lecturer Horia Tudor Stanca, MD, PhD, FEBO** **Chief of the Eye Clinic, “Prof. Dr. Agrippa Ionescu” Clinical Emergency Hospital, Bucharest ** **Ophthalmology Department, “Carol Davila” University of Medicine and Pharmacy Bucharest**

